# A review of the evolution and trajectory of the African union as an instrument of regional integration

**DOI:** 10.1186/2193-1801-3-101

**Published:** 2014-02-19

**Authors:** Innocent EW Chirisa, Artwell Mumba, Simbarashe O Dirwai

**Affiliations:** Department of Rural & Urban Planning, University of Zimbabwe, PO Box MP167, Mt Pleasant, Harare, (263-4) Zimbabwe

**Keywords:** Independence, Unity, African integration, Decolonisation, Peace, Development

## Abstract

This review paper seeks to analyse African integration in terms of its magnitude of solidarity, the state and typology of integration and functioning. It assesses the strengths, weaknesses, objectives, successes and failures of the African integration project as well as threats to its survival. The primary goal is to sift between issues with the view of better informing the future of the integration. The paper acknowledges how, in 2002, the OAU (formed in 1963) convened to reconstitute and become the African Union (AU) composed of eight Regional Economic Communities. The reformed union has spelt out gender equality, strategic planning, intra-trade, non-indifference to suffering in member states and sustainability, as additional objectives to those of the former OAU. This idea has been to foster integration to promote peace, security and cooperation hence solidarity. It can now be assessed succinctly that African integration has arisen in the need for amalgamation of efforts to solve African problems with African solutions.

## Introduction

There are current calls for Africa to define itself culturally, politically, and economically. No doubt, this is the same space in which innovative and technological developments characterising today’s world must also be embraced (Akande 2010). These calls are premised on Africa’s ability to self-identify in terms of mapping its own capacities and limitations (Wazama 2001; Wapmuk 2009). The 1884/1885 Berlin Conference that saw the partitioning of Africa among European countries embarking on imperialism may no doubt, have ‘sowed the weeds’ of African paralysis along divisional and ethnic lines, a divide-and rule practice. Kwame Nkrumah of Ghana, Julius Nyerere of Tanzania and Nasser of Egypt were radical evocative voices for Africa to have the Organisation of African Unity (OAU) to try and iron out these differences, while also integrating the people and economies of Africa as a region. This paper seeks to analyse African integration in terms of its magnitude of solidarity, and the state and typology of integration and functioning. It assesses the strengths, weaknesses, objectives, successes and failures of the African integration project as well as threats to its survival. The primary goal is to sift between issues with the view of better informing the future of the integration. The paper begins by outlining the origins of the Organisation of African Unity (OAU), and then tells how the same has transformed itself into the African Union (AU). Furthermore it explains the theoretical underpinnings of regional integration, and then heads on to provide a detailed analysis of the efforts of regional integration in Africa. This is done by way of critical review and analysis. Africa’s regional integration is primarily aimed to tackle the challenges experienced by member states in the rapidly changing world (Okhonmina 2009). The other reason is to create a platform for strategic political and economic alliances. Respective unions like the European Union (EU), North American Free Trade Area (NAFTA) and Association of South East Asian Nations (ASEAN) provide common markets that foster avenues for trade creation and diversion when needed. In this strand, Africa had no option but to follow the trend and cooperate. Africa seeks to develop from home-formulated ideas (Palmer 2007: 1).

In 1963, a group of independent African countries came together to form an integrated union. The union was named the Organization of African Unity (OAU). Its aims were spelt out explicitly as: decolonising Africa, protecting sovereignty, achieving global competence and attaining socio-economic transformation (Department of Foreign Affairs 2004). African integration can refer to desired and extant political, economic, cultural, social and geographical cohesion among the people and nations of Africa that instil a sense of belonging. In the nuances of political ideology, it has sometimes been summed up in the concept and philosophy of ‘Pan-Africanism’ (cf. Nkrumah 1963; Steinberg 2001). Kwame Nkrumah, the first black president of Ghana advocated for the formation of a United States of Africa (USA). This as he saw it, would see African nations coming together into a single government that would work for the realisation of equity in resource allocation in a bid to create a stronger force to vehemently compete in the world market and hence result in their voice being heard (Okhonmina 2009). Julius Nyerere, the first president of the Republic of Tanzania also believed in the same but differed methodologically. For Nyerere, the United States of Africa could only be achieved as a gradual process. In the Pan-African meeting in Cairo in 1960, Nyerere spelt out the realities faced by the region then. Much later, the Colonel, Muammar Gaddafi of Libya strongly believed in the same, yet other leaders remained sceptical about this idea.

As already alluded to, the OAU was formed mainly to foster and preserve unity, solidarity and continental cooperation among African states against colonialism, and to protect its member states’ lately won independence as well as to promote the self-determination of member nations (Steinberg 2001; Shinkaiye 2006). This came from the background that in the late nineteenth century, Africa fell into the hands of foreign domination which sparked a class struggle between the white imperialists hence capitalists and the African peasants and emergent working class. Cecil John Rhodes (who colonised the plateau between Zambezi and Limpopo), for example, had the dream of colonising Africa from Cape to Cairo under one leadership and taking the advantage of geographical convenience. In his insight, Rhodes considered Africa as one land which needed to fall under one hand of control. For him, the land had to be under British control alone. Although Africa has now been decolonised with respect to political foreign domination, the idea by Cecil Rhodes in the colonisation process can be inferred as an anti-type of the African integration process which still stands out today as an issue of concern. Regionalism has borne economic, political, social and cultural interaction in the form of regional cooperation, market integration, development integration and regional integration (Lee 2002:3–5). In 1991, the OAU signed the Abuja Treaty, creating the African Economic Community (AEC). The AEC has called for total integration of African economies by 2025. African countries, instead of integrating the economies in Africa, have skipped into the world economy in reality (Lee, ibid).

In 1999, the African leaders convened in Sirte, Libya, to review the OAU charter. The review meeting passed a blind eye on Gaddafi, a despot who ruled Libya for 42 years (until 2011 when he was assassinated). The gathering saw the change of the OAU name to the African Union (AU). The change was long overdue, given the ‘demands’ of the fast-changing world. With South Africa having achieved its democracy in 1994, what remained was for OAU to re-organise and remain relevant. Apparently, the OAU had served its purpose, especially of decolonising its member states. However, what was in the name change? Largely this seems to have been in keeping with the fashionableness drive - hence modelling of the OAU along the European Union (EU) spirit (Babarinde 2007:3). With few objectives of focus and little to structure, being made and prescribed, the OAU transformed itself into AU. On the 11th of July, 2000, the Constitutive Act was signed in Lome, Togo.

In 2002, in Durban, South Africa, the official inauguration of the African Union took place. The African Union is thus an institutional manifestation of the desire for integration (Okhonmina 2009). There have been a number of transformations from non-interference with internal affairs of member states to non-indifference.

### Theoretical underpinnings of African integration

Local integration is a microcosm of national integration. National integration, which is not easily interfered with, is unity or solidarity at the country level to protect its self-determination or sovereignty. A nation expects benefits to trickle to it from the organisation. From national integration is regional integration. Continental integration is merging of the regional economic communities to form one continent to be felt at the global arena. Global integration is the cooperation of continents to collectively give potential solutions to problems facing the world like climate change. Economic, political, social and environmental types of integration are interrelated and they work together at all levels namely national, regional, continental and global levels. Politics permeates into each and every sector. The above therefore displays African competence at all levels. Improvements have been made in most areas but more needs to be done still (cf. Lee 2002).

;Regional integration is the creation of economic blocks to enhance competition and diversification of commodities. It is a process by which a group of states voluntarily, and in various degrees, access each other’s markets and establish mechanisms and techniques that minimize conflicts and maximise internal and external economic, political, social and cultural benefits of their interaction Nmehielle Regional integration is the creation of economic blocks to enhance competition and diversification of commodities. It is a process by which a group of states voluntarily, and in various degrees, access each other’s markets and establish mechanisms and techniques that minimize conflicts and maximise internal and external economic, political, social and cultural benefits of their interaction Nmehielle Regional integration is the creation of economic blocks to enhance competition and diversification of commodities. It is a process by which a group of states voluntarily, and in various degrees, access each other’s markets and establish mechanisms and techniques that minimize conflicts and maximise internal and external economic, political, social and cultural benefits of their interaction Nmehielle 2003; Govender and Ngandu 2010). The concept can be categorised in terms of function and there are four basic types of integration. The first one is *regional cooperation;* that is collaboration for execution of joint projects, development of common resources, standing towards the rest of the world and joint promotion. The second one is *market integration* and it brings specialisation according to comparative advantages of regional entities hence an increase in output and trading as a group in the world. Development integration is the third. It is a response to problems created by market integration for social and economic development. The fourth type of integration is regional integration. Soomer (2003:1) has defined it simply as “… a dynamic process that entails a country’s willingness to share or unify into a larger whole.” This means the regional integration comes from voluntary action by collaborating countries. These four identified aspects in regional collaboration (regional cooperation, market integration, development integration and regional integration) have been the bases and pillars of the African integration endeavours. These, in turn are explicitly described by three interlinked parameters of neo-liberalism, globalisation and some degree of political stability that have characterised the region since the 1950s. Neo-liberalism has always hailed the market above a command economy. This has been in keeping with the postulations by Adam Smith who believed that the market is an invisible hand that is self-correcting to be functional. Closed economies make trade hard and difficult. On the other hand, globalisation has played the role of strengthening the rationale for integration for the creations of strong linkages and enhancing international trade between nation-states. Besides, globalisation has always tended to bring with it parasitic multinational corporations that have taken Africa’s wealth from its soil, leaving poverty and destitution in Africa. Instead of integration at the continental level, the African states prefer being ridden by the world directly but unintentionally growing the world at its own expense. Regional integration is not only part of the solution but also part of the problem. In addition, political instability has always created some avenue for unity and increased societal cooperation among the affected countries who have always thought of ways of managing this instability and give way to stability, harmony and peace.

Nevertheless, freeing trade has forged negative trade balances in Africa - with poverty, low growth and unemployment remaining a challenge. Instead of bringing benefits, globalisation, to a large extent, has triggered further exploitation of local resources, rendering some countries very economically weak. Political stability is the aim of integration but it is reversed by lack of cooperation by other states. Nine months from the Arab spring in January 2011, the political and economic outlook for most of Middle East Africa and North Africa (MENA) got uncertain (O’Sullivan et al. 2011). The end of corrupt regimes in Tunisia and Egypt led to the spread of revolutions in parts of North Africa. The Arab Spring dethroned three presidents namely Zine El Abine Ben Ali of Tunisia, Hosni Mubarak of Egypt and Muammar Qaddafi of Libya. After these events, perhaps, Africa has been getting a new definition in governance and all sectors of living. The National Transition Council (NTC) in Libya has sought to be recognised as a legitimate government. It entered Tripoli on 22 August 2011. The rule against unconstitutional changes of government was broken (Sturman 2011).

### Regional communities and the question of integration

There are eight regional unions in Africa. These groupings unite people of the same geographical zone to engage in free trade and other business depending on the nations’ values of commonality. Overall, regional communities in Africa are the integration arrangements which have grown over the years. There is the West African Economic and Monetary Union (WAEMU) within the ambit of Economic Community of West African States (ECOWAS) and the Economic and Monetary Union of Central Africa (CEMAC) within the proposed Economic Community of Central African States (ECCAS) region. Within the geographic area of Common Market for Eastern and Southern Africa (COMESA) there are the Southern African Customs Union (SACU) with its associated monetary union (the Common Monetary Area, CMA), the Southern African Development Community (SADC) and the East African Community (EAC). Some countries in this region are also joined with countries in the Horn of Africa in the Intergovernmental Authority on Development (IGAD) (African Union Commission 2013). By way of showing the dates when these regional economic communities were established, the regional groups are:

- The Central African Economic and Monetary Community (CAMAC) from the 1964 Customs and Economic Union of Central Africa (UDEAC).
- The East Africa Cooperation (EAC) - 1967.
- The West Africa Economic Community (CEAO) - 1972.
- The Economic Community of West African States (ECOWAS) – 1975.
- The Southern African Development Community (SADC) – 1992 from the Southern Africa Development Conference (ASADC) of 1980.
- The Union of Maghreb Arab State (UMA) – 1988.
- The West African Economic and Monetary Union (UEMOA) – 1994.
- The Common Market for East and Southern Africa (COMESA) – 1995 from the Preferential Trade Area of 1981.

These regional communities have aided integration through establishing macroeconomic convergence and unimpeded transit facilitation, reducing costs and improving overall efficiency in transportation The underlying philosophies for the development these have been informed by the contextual factors of neo-liberalism, globalisation and the quest for regional and country political stability (UNDP 1999 – see Table 1). All the regional economic communities have introduced instruments in one form or another to promote free trade amongst member countries. They have also improved integration by creating inter-country connectivity through the global revolution in telecom technology and the growing commercialization and privatization of national services (Economic Commission for Africa 2004). Integration has also been aided as shown by visible cooperation in early warning systems, agricultural research, and capacity building through knowledge sharing. The major institutionalization of the functions of some of these regional communities has manifested itself in the works of the organs of the AU. These organs are ascribed with different mandates prescribed in Constitutive Act (see Table 2). The Act commands order in the function. Hence, the general observance of this structure provides a platform for institutional integration. For instance, one contemporary aspect to regional integration has been the obsession, like all the other global regions, to achieve the millennium development goals (MDGs) (AUC, UNECA, AFDB and UNDP, MDG 2012). Table 3 is an attempt to show the ‘tracking’ of Africa in MDG performance.

**Table 1:** **Regional integration bases**

Context	Purpose	Issue	Assessment
Neo-liberalism	Calls for limited government intervention	Led in policies like IMF/World Bank Structural Adjustment Program (SAP)	Not good, it led to poverty and marginalisation
Globalisation	World linkages Interdependence	Sub Saharan Africa international trade	30% exports to global markets.
Political stability	Political stability as a pre-requisite for sound integration	Credit lines and trade	Marginality and borrowing

Sources: UNDP(1999:2).

**Table 2:** **African union structure**

Organ	Mandate
The assembly	Determines common policies
The executive council	Coordinates and makes policies
The Pan-African parliament	Implements policies
The Court of Justice	Ensures compliance with law
The Commission	Secretarial duties
The permanent representative committee	Assists the executive
The specialized technical committee	Assists the executive in substantive matters
The economic, social and cultural council	Make decisions on prevention, management and resolution of conflicts
The peace security council	
African central bank and others	Financial backing
The African monetary fund	
The African investment bank	

Source: African Union (AU) (2012).

**Table 3:** **Africa’s performance in millennium development goals**

Goal - MDGs	Status	Remarks
Goal1: Eradicate extreme poverty and hunger	Off track	• $1.25-a-day poverty in Africa (excluding North Africa) declined from 56.5% to 47.5% during 1990 – 2008
Goal 2: Achieve universal primary education	On track: net enrolment	• Average enrolment exceeds 80%
		• Issues of quality remain
		• Most countries are expected to meet completion target
Goal 3: Promote gender equality and empower women	On track	• Good progress at primary level but weak parity at secondary and tertiary levels of education
		• High representation in parliament
Goal 4: Reduce child mortality	Off track	• Declining but slowly
Goal 5: Improve maternal health	Off track	• Declining but slowly
Goal 6: Combat HIV/AIDS, malaria and other diseases	Off track	• HIV/AIDS on decline, especially Southern Africa due to behavioural change and access to antiretroviral therapy
Goal 7: Ensure environmental sustainability	On track: improved water supply	• Few countries have reforestation plans
		• Emissions minimal in most countries with little increase
		• Most countries reduced consumption of ozone-depleting substances by more than 50%

Source: UNDP (2012).

NB: ‘off track’, as denoted in Table 2 does not mean total failure.

In a way, the structure of the AU is a reinforcing factor in consolidating the function and operations of the regional community bodies described above. It acts as an administrative tool for oversight and providing direction to these communities. However, the efforts are not without challenges and constrains.

### Novel and pressing challenges

Talking of integration in an environment of distrust and division is a big challenge indeed, yet it must be embraced that integration is not static at all. Dependency on the west or east remains a dividing line defining the member states of the AU but the AU has to devise ‘African solutions to African problems.’ The globe today is seeking solutions to global challenges like climate change, financial crises and civil unrest. Africa has so much of its own problems from which also are a vast embedded opportunities. Unfortunately it tends to borrow most of the perceived solutions from beyond its borders and fails to brew these with own home-grown ones (Nkrumah 1963: 12). It is in solution finding that integration becomes imperative (Steinberg 2001). The desire of Africa is to be complete masters of their destiny.

There are political, economic and social disparities in Africa. The modelling of the African Union was along that of the European Union – somewhat a ‘copycat’. Steinberg (2001) has observed that there are overt African disparities which he has claimed are the recipe for weakening the African Union. However and generally, African integration lies in the richness of the region’s cultural diversification. Mushikiwabo (2011), the foreign minister of Rwanda has said that African conflicts are threatening peace in the region (cf. Govender and Ngandu 2010). In his remarks, peace in Africa is hard won and conflicts are obstacles to economic prosperity. Social unrests spill effects over to the rest of the continent and undermine integration. For instance, Burundi attained independence in 1961. The country has been plagued by ethnic tensions between the Tutsi minority and Hutu majority for nearly two decades now. Shortly after President Melchior Ndayane was assassinated in 1993, the wars broke out and lasted for twelve years. Three hundred thousand people lost their lives and hundreds of civilians were displaced. South Africa as AU’s representative, stood as a mediator. An inclusive government was formed in 2001 as Burundians assented to have a ceasefire. Subsequently in 2005 the Burundians voted in the first parliamentary elections. The intervention shows the commitment by both the OAU and AU to conflict resolution and peace maintenance (Peace and Security Department - African Union Commission 2013). However, whenever elections are held in Africa there is a likelihood that violence breaks out. That draws the attention of the AU and the International Community.

### The achievements of the au: a synopsis

As already highlighted, the main goals of the OAU according to the OAU Charter of 1963 focused on the defence of the sovereignty, territorial integrity and independence of African states and supporting liberation movements in the eradication of all forms of colonialism and apartheid from Africa (Oxfam International 2012). The objectives are succinctly stated in Article II of the Charter as follows:

- To promote the unity and solidarity of the African States;
- To co-ordinate and intensify their cooperation and efforts to achieve a better life for the peoples of Africa;
- To defend their sovereignty, their territorial integrity and independence;
- To eradicate all forms of colonialism from Africa; and
- To promote international cooperation, having due regard to the Charter of the United Nations and the Universal Declaration of Human Rights.

OAU achievements included promoting human and people’s rights in the continent through the establishment of the African Human Rights Commission located in Banjul. It also witnessed the decolonisation of Zimbabwe and Namibia through organising diplomatic support and channelled financial, military and logistical aid to liberation movements. In addition, it resolved boundary conflicts in North, East and Central Africa and also successfully settling these conflicts without outside intervention or interference. It also defended the sovereignty and territorial integrity of member states, most notably in the Congo where imperialism vied for disintegration to set up a puppet state and Nigeria’s civil war which threatened the Federal Republic of Nigeria. Ending apartheid and racial discrimination in South Africa, Rhodesia and the International territory of Namibia (South West Africa) and action in favour of African Refugees through the operation of a bureau for placement and education of African refugees at the OAU headquarters are laudable. There has been provision of educational and job opportunities to African refugees and the creation of a refugee status and right of asylum to be recognized to refugees by all independent African countries. Although these achievements were attained, the terrain was never smooth but at least, measured according to its set goals, there were critical areas remarkable as great achievements.

After the tenure of the OAU, the succeeding African Union (AU) now seeks to achieve greater unity and solidarity of African countries and to be a people centred institution, trying to build a partnership between governments and all segments of civil society, in order to strengthen solidarity and cohesion among the African people and to make Africans ‘both the actors and beneficiaries of the structural changes engendered by development’ (Oxfam International 2012). The AU has been a desire by African leaders to unite all people of Africa in order to face new realities of globalisation. This includes embracing and dovetailing the contribution and role in development defined by emerging powers like India, China, Russia and Brazil. These emerging powers have been a factor in the inducement of shifting power relations between the North and the South (Oxfam International 2012).

According to its Constitutive Act, the African Union is set to accelerate the political and socio-economic integration of the continent; promote peace, security, and stability on the continent as well as promote sustainable development at the economic, social and cultural levels as well as the integration of African economies. The specific objectives are:

- To achieve greater unity and solidarity between the African countries and the people of Africa and defend the sovereignty, territorial integrity and independence of its Member States whilst promoting democratic principles and institutions, popular participation and good governance,
- To accelerate the political and socio-economic integration of the continent,
- To encourage international cooperation,
- To promote and protect human and people’s rights,
- To establish the necessary conditions which enable the continent to play its rightful role in the global economy and in international negotiations,
- To promote co-operation in all fields of human activity to raise the living standards of African people,
- To advance the development of the continent by promoting research in all fields, in particular in science and technology,
- To work with relevant international partners in the eradication of preventable diseases and the promotion of good health on the continent.

Looking at the developments happening in Africa, it can be concluded that the goals of OAU/AU are not being met, especially those of the later. Africa continues to engage at the periphery of the global economy, as is evident from the continent’s declining share in global production and trade, with the majority of sub-Saharan Africa’s 47 countries being labelled small and least developed, according to UNCTAD (2007). The AU seeks to face new realities of globalisation, promote development and trade but its landlocked countries (15 Sub-Saharan countries) have high trade transaction costs, border barriers. Many other constraints exist and more generally being the high costs of doing business in Africa. Progress in African integration is mixed across sectors, regional economic communities, and member states. There have however been some strides in trade, communications, macroeconomic policy, and transport. Some regional economic communities have made significant progress in trade liberalisation and facilitation (The West African Economic and Monetary Union, or UEMOA, and the Common Market for Eastern and Southern Africa, or COMESA), in free movements of people (the Economic Community of West African States, or ECOWAS), in infrastructure (the Southern African Development Community, or SADC, and the East African Community, or EAC), and in peace and security (ECOWAS and SADC)(Economic Commission for Africa 2004).

Africa has seen a superb rise in economic growth over the decade - 2002 to 2012 (UNDP 2012). Despite continual uncertainties, among the fastest growing economies in the world are African countries. Economic growth is a perpetual process. Judging development merely on the Gross Domestic Product (GDP) can produce fallacious results. GDP barely reflects the true dynamics in economic performance. For instance, Africa is mainly based on the informal economy, which is not captured fully by the GDP lens.

Uneven food access remains a major challenge in Africa (Beddington *et al.*^2012^). Since 2000, Africa has experienced severe food shortages affecting the Sahel region of West Africa. Millions of people were affected in the Horn of Africa and eventuating in Somalia. Women and children are the worst affected (UNDP 2012). However some parts of Africa are well-fed, thereby having a surplus. African development has been reflected in the Millennium Development Goals (MDGs). Table 3 shows the status Africa’s MDG performance in 2012.

### Is integration working for Africa?

The Economic Commission for Africa (ECA) became the champion of regional integration, already in the mid-1960s, proposing the division of Africa into regions for the purposes of economic development (African Union Commission 2013). African governments have concluded a very large number of regional integration arrangements, several of which have significant membership overlap (Hartzenberg 2011). Upon independence, African leaders have embraced regional integration as an important component of their development strategies and concluded a very large number of regional integration arrangements, which are usually neighbourhood arrangements. It must be noted that the current African integration arrangements can be divided into two broad groups: those that fit into the Lagos Plan of Action (LPA) adopted in April 1980, and those that were either in existence or came about outside the LPA (African Union Commission 2013). The integration efforts in African countries have not been effective in what they were established to do. According to Hartzenberg (2011), while they are characterised by ambitious targets, they have a dismally poor implementation record. African regional integration arrangements are generally ambitious schemes with unrealistic time frames towards deeper integration and in some cases even political union (Hartzenberg 2011; Nathan 2004).

Governance challenges and financial constraints hinder progress in the societies. Both successes and failures characterise the African Union in its quest to achieve its own goals and the Millennium Development Goals (MDGs) (Murithi 2008). Security and stability are meant to promote integration, and integration to prolong peace and initiate consensus and sustainability in development (AU 2009). The AU Peace and Security Architecture (APSA) serves and protects people from attacks. Peace is ensured to quell, in prevention, any likely violations of peace such as unconstitutional coups and civil wars. After chaos the AU paves a way for civilians to come to terms. Succeeding the OAU whose major concern had been decolonisation, the AU focused on enhancing human security and consolidating democracy (Rupiya 2012). The AU intervened in Sudan where the conflict had arisen between Khartoum and Juba after the Comprehensive Peace Agreement (CPA) of 2005 and eventually this resulted in a new state of South Sudan on 9 July 2011. The African Union could not achieve the peace and UN was allowed to impose sanctions if the three months from 25 April 2012 would not be respected. President Kirr of South Sudan got a US$8 billion loan to build hydroelectric infrastructure, roads, hospitals and 5 universities. In the case of Sudan, if Africa had no oneness it would find itself divided to the South Sudan and North Sudan in a war. Currently such a war is in progress and the AU has not managed to deal with the crisis (cf. Nathan 2004).

Nonetheless, the AU has been described as new wine in old bottles (Sesay 2008: 8) because of its failure to protect human rights in member states. The objectives and principles of the union, as set out in Articles 3 and 4 include clear provisions of democratic institutions, observance of human rights, and the rule of law and handling of gender issues. In Africa, in the states, there are few checks for the recognition of human rights (Shinkaiye 2006). To achieve African integration or cooperation, the Pan-African Parliament stands for the legislative role. It has to play an oversight role in actions of African executives. That role promotes checks and balances for accountability. The African Court of Justice and Human Rights and the Economic and Social Council (ECOSOCC) can contribute much accountability. As part of cause for integration, a Science and Technology Consolidated plan was formulated and launched into action in 2005. It was implemented to combat climate change and control the loss of biodiversity.

The concern is over some cases like that of funds from the US (Roach and Schaefer 2012). The US assisted the AU with $5.8 million and $258 million from 2007 to 2012 to support the African Union Mission in Somalia. These funds were sent directly to African governments instead of channelling the funds to the US Mission or AU itself. Integration entails participation. True integration is not biased in all terms including gender. The election of Nkosana Dhlamini Zuma makes history; as the first woman to lead the AU Commission, there is every hope that women participation will increase. On their own, Africans can alleviate hunger, poverty, homelessness and unemployment. There is a paradigm shift from the old notion of having the AU as a talk-shop. Although the AU registered some successes, the fulfilment was in relation to peace and security. Poverty in Africa still remains a huge challenge. The 2009 – 2012 Strategic Plan has not been implemented with consistency and accountability. This has emanated from weak integration of the African Union Commission and RECs as well as poor monitoring and evaluation systems. All these problems are coming from faulty governance. So it will be difficult to meet the Millennium Development Goals. Climate change again needs to be addressed and be adapted to as it deteriorates standards of living. At the 19th Summit, the now ousted President Mohamed Morsi of Egypt remarked that trade and investment cooperation will see Africa develop.

## Conclusion and lessons learnt

From the foregoing paragraphs, the following lessons can be drawn in the discourse of African integration - that peace, security and prosperity of AU nations and states are embedded in integration. Regional communities in Africa can bring about the much needed economic development and that true integration must embrace globalisation as a real phenomenon of great influence to development. In addition, a common definition of democracy is required as the recent uprisings against African leadership have come due to the demand for freedom as the benefits of development tending to accrue to a few at the expense of the majority. Moreover, the world is fast transforming, so African integration must not be static. The region should not treat itself as a closed system that has no effect to the world and that cannot be influenced by the world. Change of names and places of inaugurations does not necessarily mean ideological transformation (Sesay 2008: 7). It is important to appreciate achievements made by the AU. It is prudent to face reality. On its way, Africa has been reactive to the challenges such as alliances in the world and conflicts. Now there are problems, opportunities and threats in Africa that cannot be ignored. Those are the determinants of the future of Africa. A self-determined future is possible and can be facilitated by the AU; all Africans play their critical roles for the better, so switching from the culture of reactivity to pro-activity should be a reality. The AU has succeeded in bringing down colonialism. There remains challenges in gender equality, human rights, non-indifference and socio-economic development as enshrined in the Union’s Constitutive Act. Proper planning, accountability, sovereignty, democracy, public participation, intra-trade, global participation, rule of law, integration at regional level, integration among organs, sustainability and reforms for social, economic, political, cultural and spatial development remain ideal and rather romantic. Africa being largely an agro-based economy requires that technology be fully embraced in general primary production sector.
